# XRN2 interactome reveals its synthetic lethal relationship with PARP1 inhibition

**DOI:** 10.1038/s41598-020-71203-7

**Published:** 2020-08-28

**Authors:** Praveen L. Patidar, Talysa Viera, Julio C. Morales, Naveen Singh, Edward A. Motea, Megha Khandelwal, Farjana J. Fattah

**Affiliations:** 1 Department of Chemistry, New Mexico Institute of Mining and Technology, 801 Leroy Pl., Socorro, NM 87801 USA; 2 Department of Neurosurgery, University of Oklahoma Health Science Center, Oklahoma City, OK 73104 USA; 3 Department of Biochemistry and Molecular Biology, Simon Comprehensive Cancer Center, Indiana University School of Medicine, Indianapolis, IN 46202 USA; 4 Simmons Comprehensive Cancer Center, University of Texas Southwestern Medical Center, Dallas, TX 75390 USA

**Keywords:** Cancer, Cell biology, Molecular biology

## Abstract

Persistent R-loops (RNA–DNA hybrids with a displaced single-stranded DNA) create DNA damage and lead to genomic instability. The 5′-3′-exoribonuclease 2 (XRN2) degrades RNA to resolve R-loops and promotes transcription termination. Previously, XRN2 was implicated in DNA double strand break (DSB) repair and in resolving replication stress. Here, using tandem affinity purification-mass spectrometry, bioinformatics, and biochemical approaches, we found that XRN2 associates with proteins involved in DNA repair/replication (Ku70-Ku80, DNA-PKcs, PARP1, MCM2-7, PCNA, RPA1) and RNA metabolism (RNA helicases, PRP19, p54(nrb), splicing factors). Novel major pathways linked to XRN2 include cell cycle control of chromosomal replication and DSB repair by non-homologous end joining. Investigating the biological implications of these interactions led us to discover that XRN2 depletion compromised cell survival after additional knockdown of specific DNA repair proteins, including PARP1. XRN2-deficient cells also showed enhanced PARP1 activity. Consistent with concurrent depletion of XRN2 and PARP1 promoting cell death, XRN2-deficient fibroblast and lung cancer cells also demonstrated sensitivity to PARP1 inhibition. XRN2 alterations (mutations, copy number/expression changes) are frequent in cancers. Thus, PARP1 inhibition could target cancers exhibiting XRN2 functional loss. Collectively, our data suggest XRN2’s association with novel protein partners and unravel synthetic lethality between XRN2 depletion and PARP1 inhibition.

## Introduction

Preserving genomic integrity is critical for ensuring survival and faithful transmission of genetic information. Compromised genomic integrity results in several debilitating diseases such as neurodegenerative disorders and cancer^1,2^. The nucleic acid structures containing RNA–DNA hybrids with a displaced single-stranded DNA (R-loops) are generated in cells during transcription and their persistence leads to DNA damage and genomic instability^3,4^. One of the major sources of R-loops is impaired regulation of RNA polymerase II (RNAPII) at transcription termination sites of protein coding genes. In humans, 5′-3′-exoribonuclease 2 (XRN2) is one of the several factors that promotes RNAPII transcription termination^5^.

XRN2 is a highly conserved protein, and its homologs in budding yeast, fission yeast, and mice are known as Rat1, Dhp1, and Dhm1, respectively^6–8^. At a molecular level, the XRN2 protein processively degrades downstream cleaved RNAPII-associated RNA to promote termination, and its ribonuclease activity is essential for genome-wide poly(A) site-dependent RNAPII termination^9,10^. The XRN2 protein is also known to promote co-transcriptional degradation of aberrant pre-mRNA^11^. XRN2 physically interacts with pre-mRNA 3′ processing factors p54(nrb) and PSF, which in turn facilitates pre-mRNA 3′ processing and transcription termination^12^. Another termination factor known as Kub5-Hera (K-H/RPRD1B) also associates with XRN2 to facilitate its recruitment to termination sites^13^. The XRN2 protein has also been shown to associate with mRNA decapping factors to facilitate coupled decapping of nascent transcripts and premature termination to restrict bidirectional RNAPII elongation^14^. Furthermore, XRN2 induces premature termination in a microprocessor-dependent manner and is involved in limiting the levels of promoter-associated non-productive transcripts^15,16^. XRN2 is also involved in general RNA degradation, gene silencing, and rRNA maturation^17–19^.

In general, XRN2’s role is well understood in RNA metabolism; however, its impact on other cellular processes, especially in DNA repair and replication, is poorly understood. A recent study highlights XRN2’s involvement in DNA damage response and replication stress, and provides evidence that XRN2 depletion leads to genomic instability in immortalized fibroblast as well as cancer cells^20^. Moreover, genetic alterations in XRN2 including mutation, copy number variation, and mRNA expression are frequent in a variety of cancers^21,22^. Also, XRN2 polymorphism has been linked to an increased risk of lung cancer^23^. It is conceivable that expression loss or mutations compromising XRN2 function could promote DNA damage, elevate replication stress, enhance genomic instability, and contribute to carcinogenesis. Therefore, defining the cellular function of XRN2 in processes besides RNA metabolism is critical to build a mechanistic understanding of the complex phenotypes that arise after XRN2 deficiency and identify potential avenues for targeting XRN2 vulnerabilities in cancer.

To systematically explore XRN2’s novel cellular functions, we utilized a combination of proteomics, bioinformatics, biochemical, and biological approaches. Similar strategies have been successfully implemented in elucidating the role of another transcription termination factor, Kub5-Hera, in cellular processes besides RNA metabolism^24^. We screened XRN2-associating proteins using tandem affinity purification-mass spectrometry and employed bioinformatics and biochemical approaches to validate these interactions. Using multiple complementary approaches, we found that XRN2 associates with several proteins involved in cellular processes separate from RNA metabolism. Major pathways related to XRN2 include cell cycle control of chromosomal replication and DSB repair by non-homologous end joining (NHEJ). The XRN2 protein associates with DNA repair proteins (Ku70, Ku80, DNA-PKcs, PARP1), DNA replication proteins (MCM2-7, PCNA, RPA1), and RNA metabolic proteins (RNA helicase proteins, PRP19, p54(nrb), and splicing factors). XRN2-depleted cells showed compromised survival after additional knockdown of specific DNA repair proteins, including PARP1. These cells also showed elevated PARP1 activity and sensitivity to PARP1 inhibition demonstrating a synthetic lethal relationship between XRN2 depletion and PARP1 knockdown/inhibition. XRN2 alterations (mutations, copy number/expression changes) are frequent in a variety of cancers. Thus, PARP1 inhibitors could potentially target cancers exhibiting deficiency in XRN2 function. Collectively, our data suggest XRN2’s association with several novel protein partners involved in diverse cellular processes and unravel a synthetic lethal relationship between XRN2 depletion and PARP1 inhibition.

## Results

### Identification of XRN2-associating proteins, processes, and pathways

**Figure 1:**
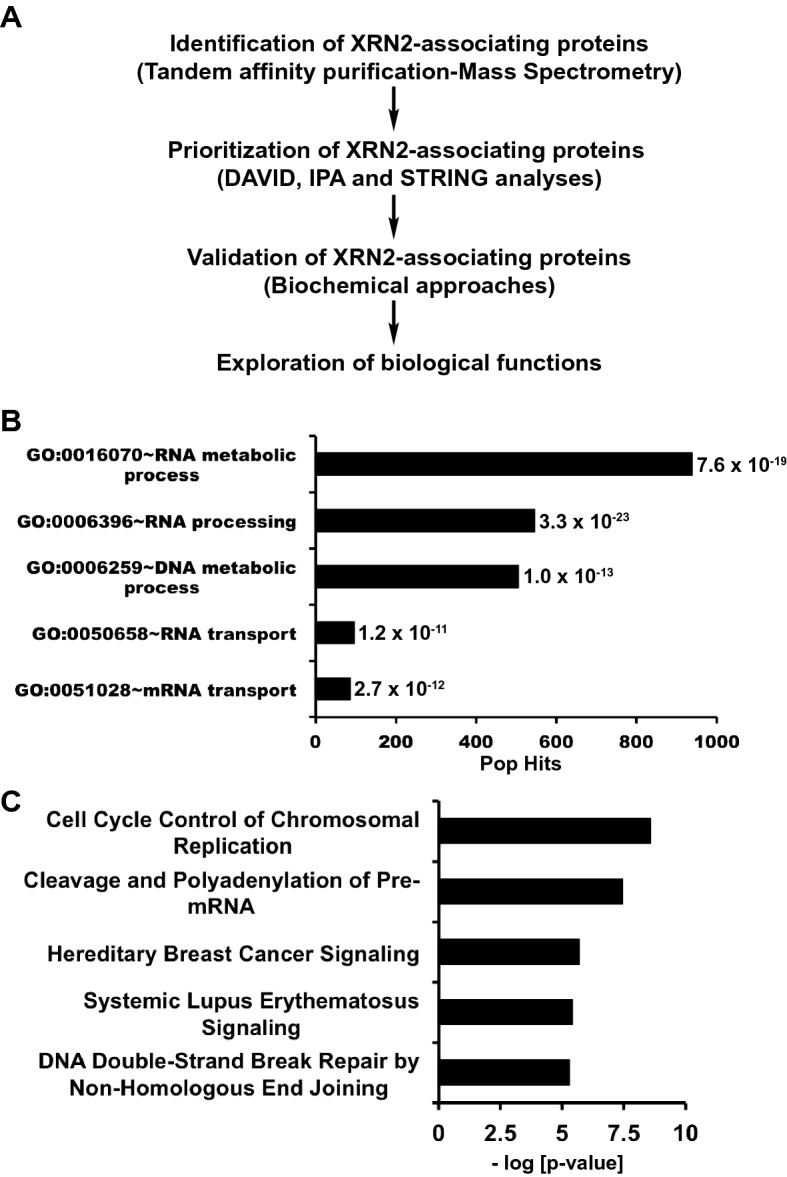
Identification and prioritization of XRN2-associating proteins. (**A**) General outline of the strategy used to identify XRN2-assocating proteins. (**B**) The top five categories of proteins associated with XRN2 obtained from DAVID analysis. List of proteins obtained from tandem affinity purification-mass spectrometry analyses of TAP-XRN2 was used as an input. Bar graph represents Gene Ontology (GO) term and corresponding population hits (Pop hits) with indicated *p*-values. (**C**) The top five canonical pathways related to XRN2 obtained from Ingenuity Pathway Analysis (IPA) with input described above. Bar graph represents pathways plotted against corresponding – log [*p*-value].

The role of XRN2 is well understood in RNA metabolic processes including transcription termination and RNA processing^25^; however, little is known about its involvement in other cellular processes. Therefore, we devised a strategy that combines proteomics, bioinformatics, biochemical, and biological approaches to systematically investigate the cellular roles of XRN2 in processes beyond RNA metabolism. A general schematic of this strategy is presented here (Fig. 1A). Tandem affinity purification coupled with mass spectrometry (TAP-MS) analyses of XRN2 (Fig. S1A,B) revealed the identity of a large number of proteins isolated from TAP-XRN2 pull-downs and subjected to further filtering using multiple criterions, as described in detail under the “Methods” section. TAP-empty vector pull-downs were included as negative controls. A final list of proteins that is representative of three biological replicates and satisfied stringent filtering is presented here (supplementary data, Table S1).

To add another layer of filtering, we utilized several bioinformatics tools. Functional categorization of XRN2-associating proteins was performed via DAVID analyses, and Gene Ontology (GO) terms were plotted against population hits (Pop Hits) with respective *p*-values, revealing the top five biological processes related to XRN2, including RNA and DNA metabolic processes (Fig. 1B and Table S2). Inclusion of RNA metabolic processes is consistent with large body of literature describing XRN2’s importance in RNA metabolism including transcription termination^25^. Further evaluation of XRN2-associating proteins using Ingenuity Pathway Analysis (IPA), where canonical pathways were plotted against respective –log [*p*-values], provided the top five canonical biological pathways relevant to XRN2, including cleavage and polyadenylation of pre-mRNA, cell cycle control of chromosomal replication, and DNA double strand break (DSB) repair (Fig. 1C and Table S3). Consistent with the current study, XRN2’s role in DNA repair and control of replication is supported by a recent study reported by our group^20^. Our screening data consisted of well-known XRN2 binding partners including RNAP II, NONO (p54(nrb)), PSF, Kub5-Hera (K-H/RPRD1B), p15RS (RPRD1A), Ku80 and other proteins^12,20^. We also identified several novel XRN2-associating proteins including PARP1, RPA1, PCNA, PRP19, CDK1, DDX1, SSBP1, DDB1, etc. Some of these proteins are also present in the BioGrid database as XRN2-interacting partners (https://thebiogrid.org). Collectively, the TAP-XRN2 screening data and bioinformatics analyses suggest that the strategy we devised to explore biological roles of XRN2 is effective, consistent with known functions of XRN2, and revealed several novel interactions.

### Validation of XRN2-associating proteins

**Figure 2:**
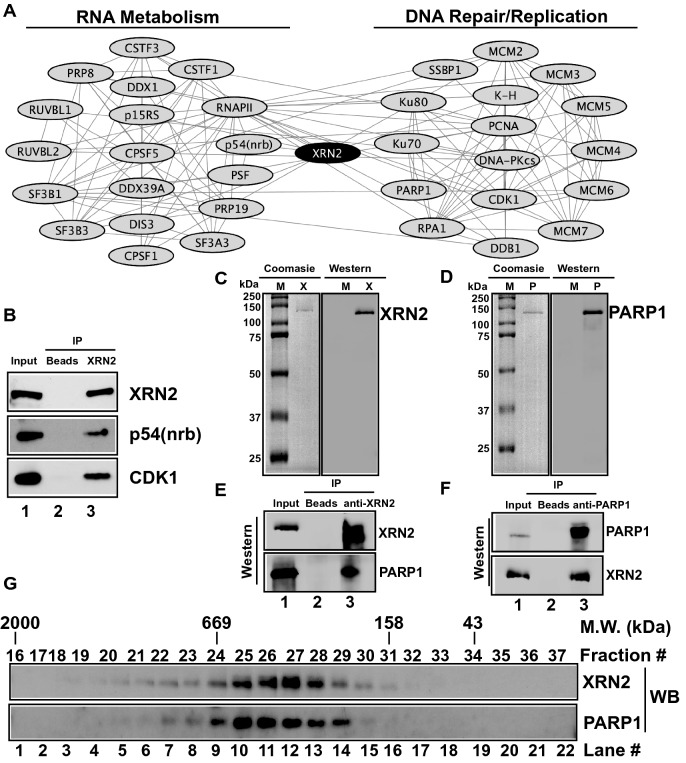
Validation of XRN2-associating proteins. (**A**) Selected proteins involved in RNA metabolism, DNA repair, and DNA replication were used to generate a network of proteins associated with XRN2 using STRING 9.1 database (https://string-db.org) and Cytoscape 3.4.0. (https://cytoscape.org). Each single line connecting individual proteins represents a connection between given proteins. (**B**) Co-immunoprecipitation of TAP-XRN2 with indicated associating proteins. The XRN2 pull-down was carried by specific antibody against it and all the proteins were detected by Western blot analyses. Lane 1 represents input for IP and lanes 2 and 3 represent IP by beads and anti-XRN2 antibodies, respectively. Note that co-IP data validates XRN2’s association with novel proteins of interest. (**C**) SDS-PAGE for purified XRN2 stained with coomassie (left) and validated with Western blotting (right) using anit-XRN2 antibody. (**D**) SDS-PAGE for purified PARP1 stained with coomassie (left) and validated with Western blotting (right) using anti-PARP1 antibody. (**E**) Co-immunoprecipitation of purified XRN2 and PARP1. The XRN2 immunoprecipitation (IP) was carried by anti-XRN2 antibody and proteins were detected by Western blot analyses. (**F**) Co-immunoprecipitation of purified XRN2 and PARP1. The PARP1 IP was carried by anti-PARP1 antibody and proteins were detected by Western blot analyses. Lane 1 represents input for IP and lanes 2 and 3 represent IP by beads and anti-XRN2 (**E**) or anti-PARP1 (**F**) antibodies, respectively. Note that these Co-IP data using purified proteins strongly suggest a direct interaction between XRN2 and PARP1. M; molecular weight marker in kilo Dalton (kDa), X; XRN2, and P; PARP1. Western blots are representative of at least three independent experiments. (**G**) Nuclease-treated HeLa whole cell lysate was subjected to gel filtration chromatography. The presence of XRN2 and PARP1 in given fractions were detected via Western blotting using specific antibodies. Note that co-elution of XRN2 and PARP1 further suggest the complex formation between these two proteins. WB; Western blot. Raw data for all the Western blot images are provided in the supplementary information.

We generated a protein–protein interaction network for certain XRN2-associating proteins using STRING analyses that included proteins satisfying all the above-mentioned filtering (Fig. 2A). Indicated proteins were included in STRING analyses based on our primary interest in factors related to RNA metabolism, DNA repair, and replication (Fig. 2A). Next, we decided to validate some of these interactions using biochemical approaches, including co-immunoprecipitation and gel filtration chromatography. Protein extracts from 293 T TAP-XRN2 cells were used to pull-down XRN2 along with its associating proteins using XRN2-specific antibodies and proteins were detected using Western blotting. Pull-downs from beads containing no antibody were used as negative controls and the known XRN2-associating protein, p54(nrb), was used as positive control. With an XRN2-specific antibody, we observed appreciable pull-down of XRN2, its known binding partner (p54(nrb)), and a novel associating protein (CDK1), where CDK1 was not detected in pull-downs from beads without antibody (Fig. 2B).

The presence of classical non-homologous end joining (C-NHEJ) factors Ku70 and Ku80 in TAP-XRN2 pull-downs and further support of these interactions in bioinformatics analyses were anticipated (Table S1, Fig. 2A) since we showed interaction of these proteins with XRN2 in our previous study^20^. However, the presence of alternative NHEJ (alt-NHEJ) factor PARP1 in our screen was intriguing. Therefore, to further evaluate this interaction, we decided to use recombinant XRN2, recombinant Flag-PARP1, anti-XRN2 and anti-PARP1 antibodies to carry similar co-immunoprecipitation as described above. First, SDS-PAGE and Western blotting were used to determine the purity of XRN2 and PARP1 (Fig. 2C,D, respectively). Next, we tested the physical interaction of XRN2 with PARP1 using XRN2 antibody. Indeed, we pulled-down PARP1 along with XRN2 using anti-XRN2 antibody (Fig. 2E lane 3), where control beads did not show any signal for these proteins (Fig. 2E lane 2). In a reciprocal experiment, we pulled-down PARP1 using anti-PARP1 antibody, and as expected, the PARP1 pull down was accompanied with XRN2 (Fig. 2F lane 3) and control beads did not show any signal for these proteins (Fig. 2F lane 2). Together, pull-down studies with purified proteins strongly indicate a physical interaction between XRN2 and PARP1. Moreover, co-elution of XRN2 and PARP1 in gel filtration chromatography analyses of nuclease-treated cell lysates also indicated that they could form a higher-order complex in cells (fraction #24–29 in Fig. 2G). Collectively, these results provide biochemical evidence to our screening data and further support some of the known and novel interactions that we have identified.

### Concurrent depletion of XRN2 and specific DNA repair proteins compromised cell survival

**Figure 3:**
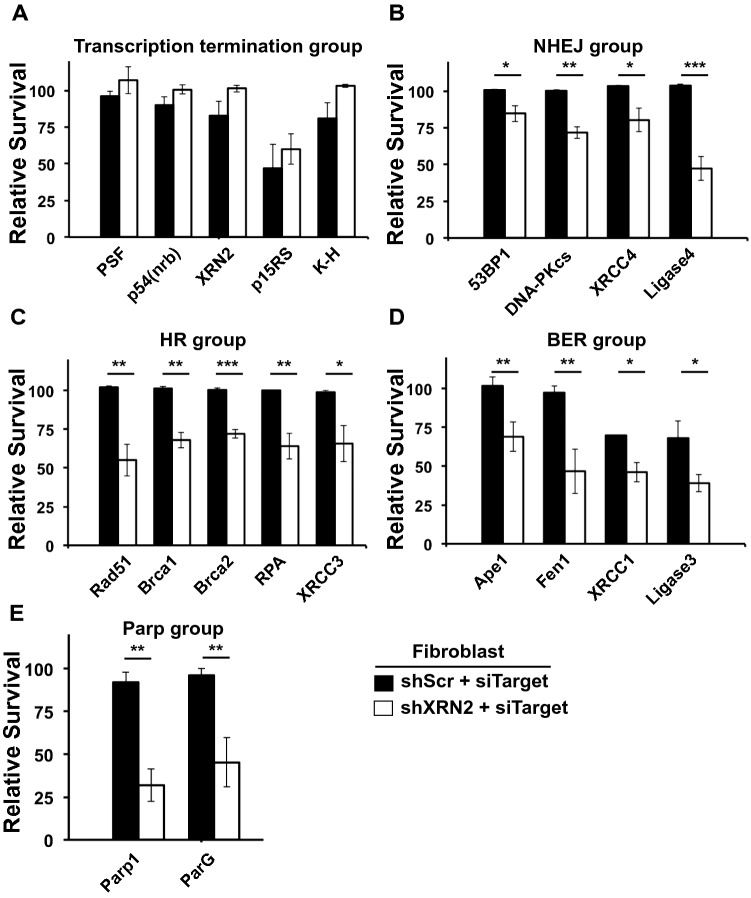
XRN2 knockdown cells display compromised survival with additional depletion of DNA repair proteins. (**A**–**E**) Relative cell survival analyses of shScr and shXRN2 cells after siRNA-mediated additional knockdown of transcription termination factors (**A**), Non-homologous end joining (NHEJ) factors (**B**), homologous recombination (HR) factors (**C**), base excision repair (BER) factors (**D**), and PARP1 and PARG (**E**). Graphs represent % mean ± SEM for treated/control samples from experiments performed 3 times in triplicate. *p*-values were obtained via two-tailed student’s t-tests. *p < 0.05; **p < 0.01; ***p < 0.001, comparing shScr vs. shXRN2 with indicated siTarget.

The proteomics data and bioinformatics analyses presented above clearly associate XRN2 with DNA repair and provide molecular links to DSB repair pathway. These data, in conjunction with previously published work^20^, compelled us to further define the biological role of XRN2 in DNA repair. Therefore, we conducted a focused siRNA-mediated knockdown screen in which XRN2 depleted cells (i.e., shXRN2 cells) were transiently transfected with specific siRNAs to suppress the expression of various DNA repair or transcription termination factors and followed by assessment of cell survival. The goal of this screening was to identify factors whose depletion lead to compromised cell survival in XRN2 depleted cells (i.e., identification of synthetic lethal combination(s)). Knockdown of XRN2 alone did not cause significant cell death in fibroblast cells compared to control (siScr) knockdown (Fig. S2). After double knockdown, cell survival was measured revealing that XRN2 depletion is synthetic lethal with additional depletion of several DNA repair factors involved in various repair processes including NHEJ, homologous recombination (HR) and base excision repair (BER). However, depletion of known transcription termination factors (K-H, PSF, p54(nrb), and p15RS) did not reduce the survival of shXRN2 cells (Fig. 3A). In the NHEJ group, 53BP1, DNA-PKcs, XRCC4 and Ligase4; in the HR group, Rad51, Brca1, Brca2, RPA and XRCC3, and in the BER group, Ape1, Fen1, Xrcc1, and Ligase3 were found to be synthetic lethal with XRN2 depletion (Fig. 3B–D, respectively). Interestingly, we observed that depletion of PARP1 and PARG also compromised cell survival of shXRN2 cells (Fig. 3E). These data suggest that cellular stress created by XRN2 depletion engages multiple DNA repair proteins in attempts to eliminate stress (i.e., termination defects, R-loops, DSBs, and replication stress, etc.) consequently promoting cell survival. Thus, stress induced by XRN2 depletion is transient and inadequate to promote lethality (Fig. S2); however, combining XRN2 depletion with the knockdown of specific DNA repair factors creates sustained stress ensuring lethality. Nonetheless, our results identified several specific DNA repair factors that compromise cell survival when depleted in combination with XRN2 knockdown, which included factors such as PARP1 that can be exploited for targeted therapy.

### XRN2 depleted cells exhibit enhanced PARP1 activity

**Figure 4:**
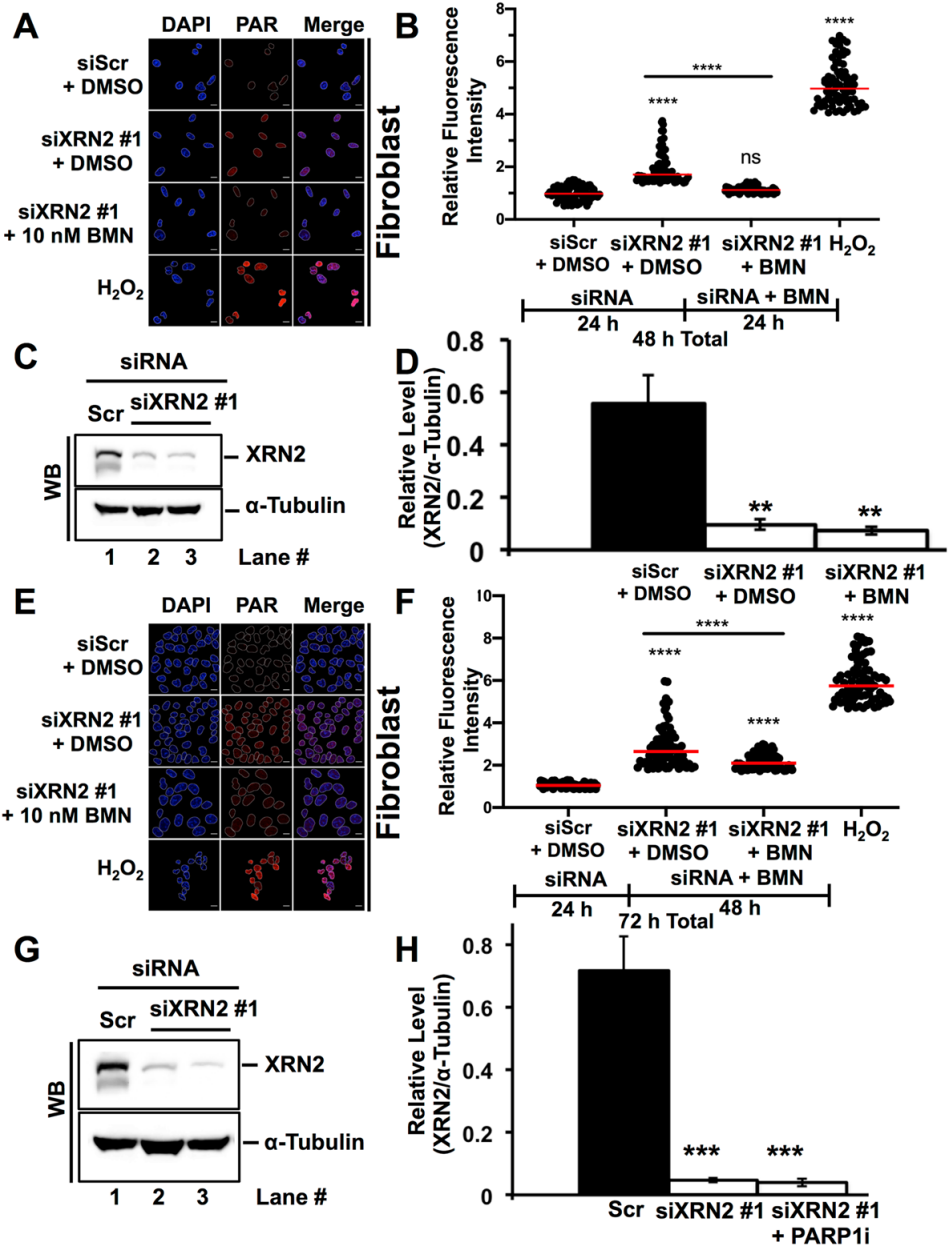
XRN2 depletion enhances PARP1 activity. (**A**) Representative confocal fluorescence microscopy images of nuclei stained with DAPI (in blue) showing PAR (poly(ADP-ribose)) immunostaining (in red) of cells treated with siScr + DMSO or siXRN2 #1 + DMSO or siXRN2 + 10 nM PARP1i (BMN 673 in DMSO). Cells treated with H_2_O_2_ (1 mM for 15 min) served as positive control. The siScr or siXRN2 #1 depletion was carried out for 48 h and BMN 673 (10 nM) treatment was carried out for 24 h as indicated in (**B**). Scale bar is 10 µm. (**B**) Quantification of fluorescence signal obtained from images shown in (**A**) and processed by ImageJ. Graph represents fluorescence intensities of nuclei of 75 individual cells (n = 3) and normalized to siScr PAR. (**C**,**D**) Representative Western blot image (**C**) and quantification (**D**) showing successful knockdown of XRN2 in cells that were used in (**A**). As a loading control, α-tubulin was used. (**E**) Representative confocal fluorescence microscopy images of nuclei stained with DAPI (in blue) showing PAR immunostaining (in red) of cells treated with siScr + DMSO or siXRN2 #1 + DMSO or siXRN2 + 10 nM PARPi (BMN 673 in DMSO). Cells treated with H_2_O_2_ (1 mM for 15 min) served as positive control. The siScr or siXRN2 #1 depletion was carried out for 72 h and BMN 673 (10 nM) treatment was carried out for 48 h as indicated in (**F**). Scale bar is 10 µm. (**F**) Quantification of fluorescence signal obtained from images shown in (**E**) and processed by ImageJ. (**G**,**H**) Representative Western blot image (**G**) and quantification (**H**) showing successful knockdown of XRN2 in cells that were used in (**E**). As a loading control, α-tubulin was used. For statistical analysis relevant to (**B**) and (**F**), one-way ANOVA was performed comparing siXRN2 #1, siXRN2 #1 + PARPi, and the H_2_O_2_ positive control means to the siScr control or siXRN2 #1 to siXRN2 #1 + PARPi. (ns, not significant; ****p < 0.0001). Raw data for all the Western blot images are provided in the supplementary information.

The synthetic lethal relationship between XRN2 depletion and PARP1 depletion was intriguing since these two proteins were found to associate with each other (Fig. 2 and Table S1). It is conceivable that DNA damage response emanating from XRN2 depletion^20^ could up-regulate PARP1 activity, which in turn promote cell survival. Therefore, cells might rely on elevated PARP1 activity to survive in the absence of XRN2. Thus, simultaneous deficiency of PARP1 and XRN2 could confer cell death. To test this notion and to probe the molecular basis of this synthetic lethality, we investigated whether XRN2 depletion alters PARP1 activity via monitoring PAR (poly(ADP-ribose)) formation through immunofluorescence confocal microscopy. Cells treated with H_2_O_2_ were utilized as a positive control. Indeed, XRN2 depleted cells (using two different time points, i.e., 48 and 72 h after knockdown) showed significant elevation of PARP1 activity when compared to the control (i.e., scramble, siScr) knockdown (Fig. 4A–H). Importantly, XRN2 depleted cells treated with the most potent PARP1 inhibitor, talazoparib (BMN 673)^26,27^ showed PAR levels significantly reduced compared to siXRN2 knockdown alone (Fig. 4A,B,E,F). Together, these data support the notion that XRN2 depleted cells exhibit elevated PARP1 activity that could promote cell survival.

### XRN2 depletion sensitizes cells to PARP1 inhibition

**Figure 5:**
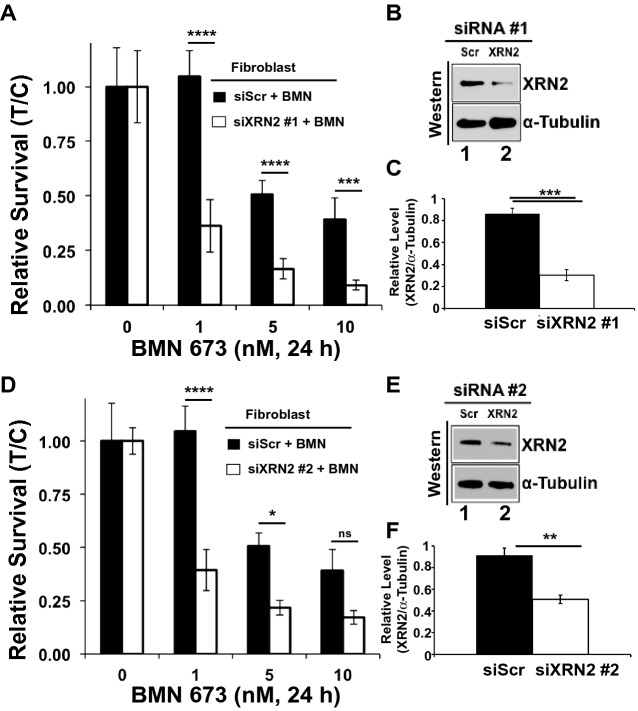
XRN2 knockdown sensitizes cells to PARP1 inhibitors. (**A**) Relative survival in the presence of indicated nM concentration of PARP1 inhibitor (BMN 673) post control (Scr) or XRN2 depletion using siXRN2 #1. (**B**,**C**) Representative Western blot image (**B**) and quantification (**C**) showing successful knockdown of XRN2 in cells that were used in (**A**). (**D**) Relative survival in the presence of indicated nM concentration of PARP1 inhibitor (BMN 673) after control (Scr) or XRN2 depletion using siXRN2 #2. (**E**,**F**) Representative Western blot image (**E**) and quantification (**F**) showing successful knockdown of XRN2 in cells that were used in (**D**). Cell survival graphs represent mean ± SEM for mock vs. drug treated (T/C) siScr and siXRN2 cells from experiments performed at least 3 times in triplicate and two-way ANOVA with Dunnett's multiple comparisons test, comparing siScr + BMN to siXRN2 #1/2 + BMN, was used to obtain *p*-value. ns, not significant; *p < 0.05; **p < 0.01; ***p < 0.001, and ****p < 0.0001. Raw data for all the Western blot images are provided in the supplementary information.

Genetic silencing of PARP1 in XRN2 depleted cells resulting in compromised survival (Fig. 3) and enhanced PARP1 activity (Fig. 4) raised a possibility of sensitivity of these cells to pharmacological inhibition of PARP1. Therefore, we sought to investigate whether XRN2 depleted cells are sensitive to the most potent PARP1 inhibitor, talazoparib (BMN 673)^26,27^. Indeed, we observed significant sensitivity of XRN2 depleted cells (using two different siRNAs) against several concentrations of BMN 673 compared to control (i.e., scramble, siScr) knockdown (Fig. 5A–F). Notably, knockdown of XRN2 alone did not sensitize fibroblast cells (Fig. S2). These data are consistent with genetic knockdown of PARP1 promoting cell death of XRN2 deficient cells (Fig. 3) and suggest that XRN2 deficiency can be therapeutically exploited using PARP1 inhibition.

### XRN2 depletion with simultaneous PARP1 inhibition enhances DNA damage

**Figure 6:**
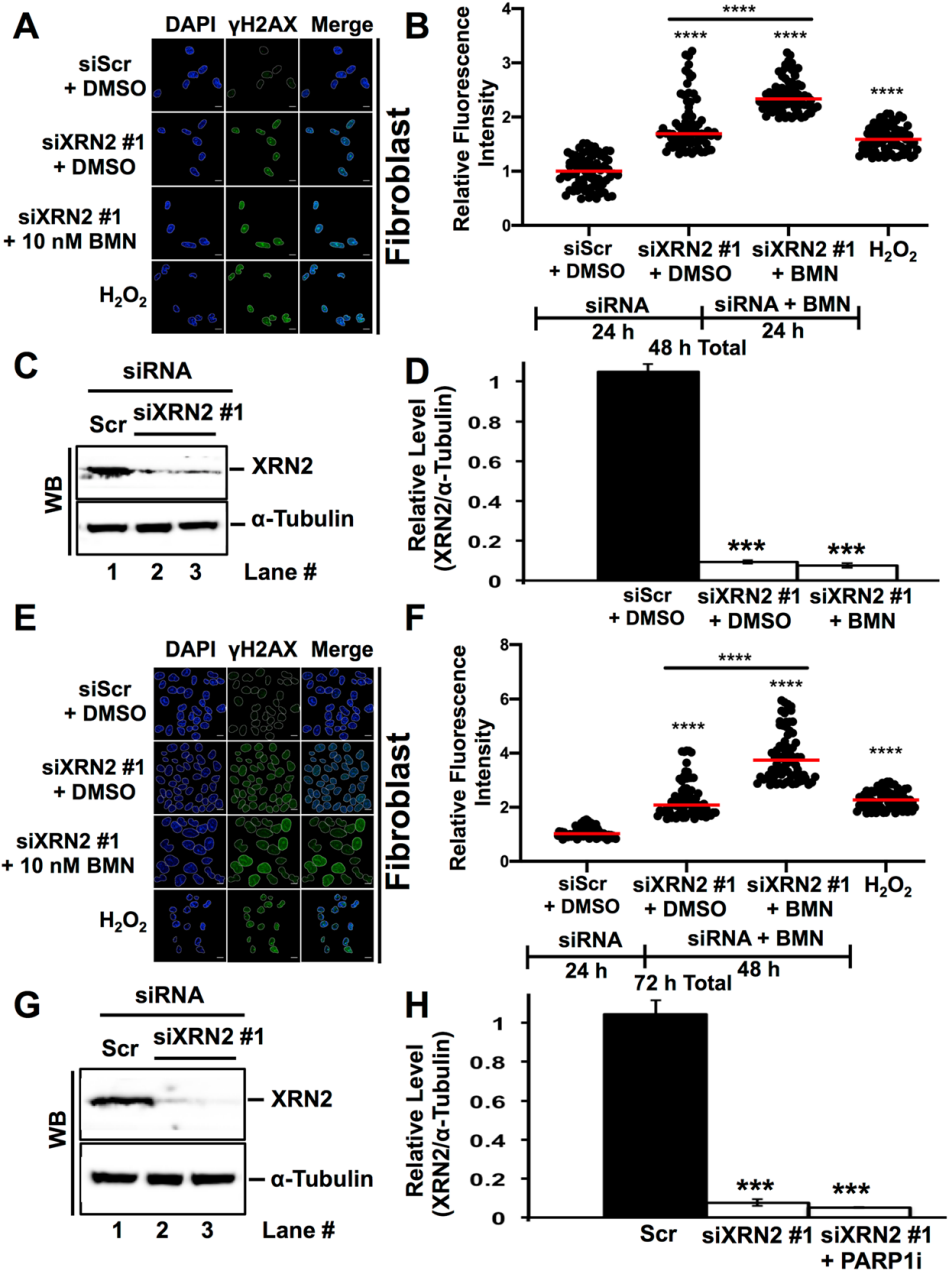
XRN2 knockdown cells treated with PARP1 inhibition display enhanced DNA damage. (**A**) Representative confocal fluorescence microscopy images of nuclei stained with DAPI (in blue) showing γH2AX immunostaining (in green) of cells treated with siScr + DMSO or siXRN2 #1 + DMSO or siXRN2 + 10 nM PARPi (BMN 673 in DMSO). Cells treated with H_2_O_2_ (1 mM for 15 min) served as positive control. The siScr or siXRN2 #1 depletion was carried out for 48 h and BMN 673 (10 nM) treatment was carried out for 24 h as indicated in (**B**). Scale bar is 10 µm. (**B**) Quantification of fluorescence signal obtained from images shown in (**A**) and processed by ImageJ. Graph represents fluorescence intensities of nuclei of 75 individual cells (n = 3) and normalized to siScr γH2AX. For statistical analysis, one-way ANOVA was performed comparing siXRN2 #1, siXRN2 #1 + PARPi, and the H_2_O_2_ positive control means to the siScr control or siXRN2 #1 to siXRN2 #1 + PARPi. (****p < 0.0001). (**C**,**D**) Representative Western blot image (**C**) and quantification (**D**) showing successful knockdown of XRN2 in cells that were used in (**A**). As a loading control, α-tubulin was used. (**E**) Representative confocal fluorescence microscopy images of nuclei stained with DAPI (in blue) showing γH2AX immunostaining (in green) of cells treated with siScr + DMSO or siXRN2 #1 + DMSO or siXRN2 + 10 nM PARPi (BMN 673 in DMSO). Cells treated with H_2_O_2_ (1 mM for 15 min) served as positive control. The siScr or siXRN2 #1 depletion was carried out for 72 h and BMN 673 (10 nM) treatment was carried out for 48 h as indicated in (**F**). Scale bar is 10 µm. (**F**) Quantification of fluorescence signal obtained from images shown in (**E**)and processed by ImageJ. Graph represents fluorescence intensities of nuclei of 75 individual cells (n = 3) and normalized to siScr γH2AX. (**G**,**H**) Representative Western blot image (**G**) and quantification (**H**) showing successful knockdown of XRN2 in cells that were used in (**E**). As a loading control, α-tubulin was used. For statistical analysis relevant to (**B**) and (**F**), one-way ANOVA was performed comparing siXRN2 #1, siXRN2 #1 + PARPi, and the H_2_O_2_ positive control means to the siScr control or siXRN2 #1 to siXRN2 #1 + PARPi (****p < 0.0001). Raw data for all the Western blot images are provided in the supplementary information.

Enhanced cytotoxicity of XRN2-deficient cells treated with PARP1 inhibitor suggested potentially higher levels of DNA damage. To test this notion and gain further insight into compromised cell survival, we utilized immunofluorescence confocal microscopy to assess phosphorylated H2AX (γH2AX) level as a DNA DSB marker. For positive control, H_2_O_2_-treated cells were used. As expected, we observed significant enhancement of γH2AX signal in XRN2 depleted cells treated with BMN 673 compared to control (i.e., scramble, siScr) and XRN2 knockdown at 48 h (Fig. 6A–D) and 72 h (Fig. 6E–H) time points. We observed discrete foci of γH2AX at 48 h time point; however, at 72 h, γH2AX staining was primarily diffused. Reason for diffused staining is unclear. Collectively, these data are consistent with the notion that XRN2 deficient cells treated with PARP1 inhibitor exhibit elevated levels of DNA damage.

### XRN2 alterations are frequent in cancers and can be targeted by PARP1 inhibition

**Figure 7:**
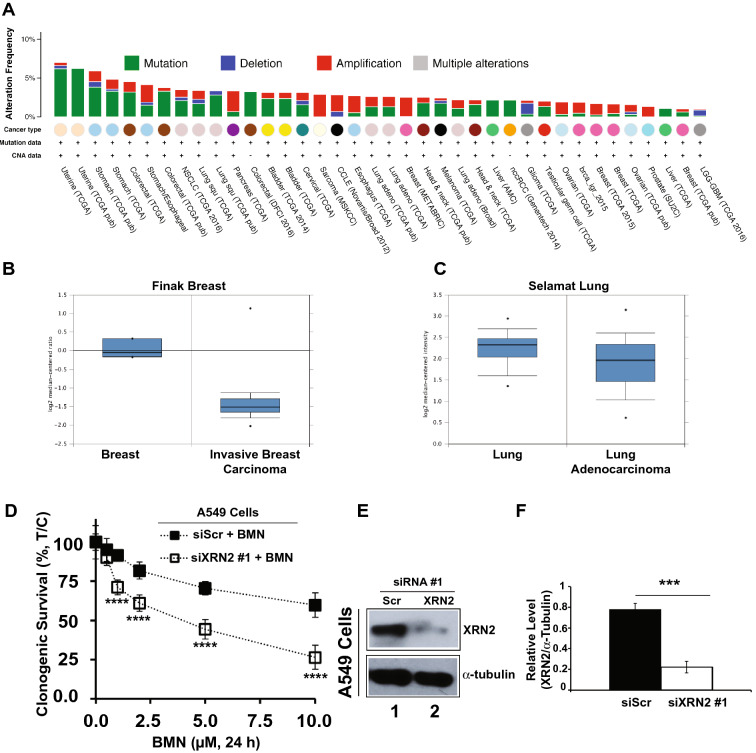
The Xrn2 alterations in various cancers can be targeted by PARP1 inhibition. Data deposited in cBioPortal and ONCOMINE by several individual studies were extracted to analyze Xrn2 alterations. (**A**) Data retrieved from cBioPortal displaying frequency of amplification, mutation, deletion, and multiple alterations (red, green, blue, and gray, respectively) in indicated cancers. Cutoffs for altered frequency and total number of samples, were set to 1% and 110, respectively. (**B**,**C**) Data retrieved from ONCOMINE for breast and lung cancer samples displaying changes in mRNA levels of the Xrn2 gene with *p*-values 1.98E−9 and 1.79E−4, respectively. (**D**) Clonogenic survival of A549 cells (lung cancer model) in the presence of indicated concentrations (µM) of PARP1 inhibitor (BMN) post control (Scr) or XRN2 depletion using siXRN2#1. Graphs represent % mean ± SEM for mock *vs*. drug treated (%, T/C) siScr and siXRN2 #1 cells from at least 3 independent experiments. For obtaining *p*-values, two-way ANOVA was used with Sidak's multiple comparisons test, comparing siScr + BMN to siXRN2 #1/2 + BMN. ns, not significant; *p < 0.05; **p < 0.01; ***p < 0.001, and ****p < 0.0001. (**E**,**F**) Western blot image (**E**) and quantification (**F**) representing successful knockdown of XRN2 in cells used in (**D**). For loading control α-tubulin was used. Raw data for all the Western blot images are provided in the supplementary information.

To explore the translational implications of findings described above, we searched XRN2 alterations in cancers by utilizing ONCOMINE and cBioPortal^21,22^. A variety of cancers showed alterations including mutations, deletions, amplifications in Xrn2 gene (Fig. 7A). We also noticed down regulation of Xrn2 mRNA expression in breast and lung cancers^28,29^. Data extracted from these studies are presented here to highlight the relevance of reduced XRN2 expression in breast and lung cancers (Fig. 7B,C, respectively).

Next, we decided to conduct a “proof of principle” study to explore the applicability of XRN2 depletion as vulnerability against PARP1 inhibition in lung cancer using A549 cell line model. Therefore, we investigated clonogenic survival of A549 cells depleted for siScr (control) or siXRN2 #1 in the presence of PARP1 inhibitor, talazoparib (BMN 673). The XRN2 depleted A549 cells showed significantly higher sensitivity to multiple concentrations of BMN 673 compared to control knockdown (Fig. 7D–F). The sensitivity against PARP1 inhibition induced by XRN2 depletion in A549 cells, however, was observed at higher concentrations (1–10 µM) of BMN 673 (Fig. 7D), compared to XRN2-depleted fibroblast cells that showed sensitivity at lower concentrations (1–10 nM) of BMN 673 (Fig. 5A,B). This difference in the extent of sensitivities is perhaps due to the intrinsic nature of these two cell lines. Nonetheless, these data strongly suggest that XRN2 vulnerabilities in cancers can be exploited by pharmacological inhibition of PARP1.

## Discussion

The role of 5′-3′-exoribonuclease 2 (XRN2) and its homologs in RNA metabolism are well documented^5–20,25^ but its function in DNA replication/repair and other cellular processes requires further investigations. Here, we devised a strategy to systematically explore XRN2’s novel biological functions and provided molecular links that associate it to specific processes and pathways. The primary conclusion of this study is that XRN2 associates with several novel factors, including PARP1, CDK1, PRP19, and RPA1, which highlights its involvement in a variety of cellular processes beyond RNA metabolism, and its deficiency renders cells vulnerable to PARP1 inhibition.

Several known XRN2-associating proteins were identified in our screening, including RNAP II, p54(nrb), PSF, Ku, RPRD1B, etc. (Figs. 1, 2 and Table S1), but we failed to detect some of the recently identified XRN2 interacting proteins including mRNA decapping factors, NF-kB repressing factor, CARF, P-TEFb, and 53BP1^14,20,30–32^. Stringent solution conditions, rigorous filtering criteria, and differential cellular context employed in this study might have resulted in elimination of some of these factors in our screening. Below, we discuss several of the validated novel XRN2-associating proteins in the context of their biological implications.

### XRN2 and CDK1

XRN2 contains consensus phosphorylation sites (pS/T-P-X-K/R) and has been shown to get phosphorylated at Thr439 that promotes its enzymatic function^31^. This consensus is used by CDKs, including CDK1, in their substrates^33^. All complementary approaches we employed here identified CDK1 as an XRN2-associating protein (Fig. 2 and Table S1). Moreover, CDK1 has been shown to phosphorylate the well-known XRN2 binding partner p54(nrb) and this phosphorylation modulates p54(nrb)’s ability to bind RNA^34^. Collectively, CDK1 association with XRN2 identified here (Fig. 2) is likely, and through phosphorylation, it may regulate XRN2 function in various processes including RNA metabolism and DNA repair/replication.

### XRN2 and DNA replication stress

The current study identified cell cycle control of chromosomal replication as one of the top biological pathways linked to XRN2 (Fig. 1). A recent study reported PRP19 recruitment to DNA damage sites via RPA to promote ATR activation, replication stress response and facilitate replication under stress conditions, and interestingly, ssDNA-coated RPA was not only found to associate with PRP19 in a pull-down assay, but also with XRN2 in this study^35^. Consistent with this finding, we observed PRP19 and RPA1 pull-down with XRN2 in all the replicates of TAP-XRN2 analyses (Fig. 2 and Table S1). Collectively, association of XRN2 with PRP19 and RPA1 is likely and raises a possibility of interplay between these proteins in regulating replication stress.

### XRN2 and DNA repair

XRN2 catalyzes degradation of RNA downstream of the poly(A)-site and ensures termination of RNAP II^9^. Likewise, deficiency of XRN2 creates transcription termination defects and leads to accumulation of R-loops^20^. However, phenotypes that arise upon XRN2 depletion are complex and include increased endogenous DNA double strand breaks (DSBs), sensitivity to DSB generating agents, compromised non-homologous end joining (NHEJ) repair, activation of ATM and ATR, replication stress and genomic instability^20^. Compromised NHEJ after XRN2 depletion^20^ and XRN2’s association with NHEJ factors (Fig. 2 and Table S1) strongly support its role in DNA DSB repair via NHEJ. Additionally, abrogation of NHEJ is emerging as a common feature due to the loss of termination factors, since deficiencies of p54(nrb), PSF, Kub5-Hera (K-H/RPRD1B), and XRN2 have been reported to compromise DSB repair^13,20,36–38^. Molecular links (i.e., Ku70, Ku80, and DNA-PKcs etc.) identified here connecting XRN2 to NHEJ now provide a firm foundation to further define the exact role of XRN2 in promoting DSB repair via NHEJ (Figs. 1, 2, 3). XRN2 associates with both Ku70 and PARP1. Ku70 promotes classical NHEJ (C-NHEJ) and PARP1 promotes alternative NHEJ (A-NHEJ) and these two proteins compete for accessing DSBs^39,40^. Since XRN2-deficient cells show compromised NHEJ^20^ and elevated PARP1 activity (Fig. 4), it is possible that XRN2 may be helping Ku70 to outcompete PARP1 for accessing DSBs. Alternatively, XRN2 may have a general role in regulating the stability of the RNA template post-NHEJ, given a recent report providing evidence that RNA serves as a template during NHEJ^41^. Moreover, RNA can stimulate PARP1 activity^42^, and in the absence of XRN2, PARP1 activity is elevated (Fig. 4). Thus, XRN2 could modulate the stability of RNA and consequently curb PARP1 activity at DSB sites. Clearly, further investigations are required to explore these possibilities.

In the absence of XRN2, elevated PARP1 activity (Fig. 4) appears to be required for cell survival because XRN2 depleted cells did not tolerate PARP1 knockdown (Fig. 3) or PARP1 inhibition (Fig. 5) and showed enhanced DNA damage (Fig. 6). However, mechanism(s) related to sensitivity of XRN2 depleted cells against PARP1 inhibition is unclear. In our previous study, depleting another transcription termination factor K-H, concomitant loss of CDK1 leading to compromised HR was responsible for sensitivity to PARP1 inhibition^43^. However, after XRN2 depletion, CDK1 levels remain unchanged, excluding this mechanism of sensitivity to PARP1 inhibition^43^. Previously, XRN2-depleted cells showed increased accumulation of HR factors at 3′-pause sites after poly(A) regions of RNAPII-driven genes and suggested that these cells up-regulate HR to resolve DSBs generated by increased R-loops formed due to XRN2 depletion^20^. Furthermore, knockdown of several HR factors led to compromised survival of XRN2-depleted cells, indicating a compensatory role of HR in the absence of XRN2 (Fig. 3C). Collectively, due to lack of clear mechanistic data, currently we do not rule out the possibility of compromised HR through some other mechanisms in XRN2 depleted cells that may explain the observed synthetic lethality. Nonetheless, association of XRN2 and PARP1 opens tantalizing possibilities of regulation imposed by these proteins on each other and how their interplay affects transcription termination, DSB repair, and resolution of replication stress. XRN2 depletion was also found to be synthetic lethal with other base excision repair (BER) factors, indicating a potential compensatory role of BER resolving stress created by the absence of XRN2 (Fig. 3D). Currently, all the possibilities mentioned above are being investigated in our laboratory.

Overall, we developed a platform for exploring novel biological roles of XRN2 that allowed us to identify novel XRN2-associating proteins, processes, and pathways (Fig. 1). Identification of known XRN2-associating proteins and processes, and the validation of few novel partners (Fig. 2), provided strength to our findings. We initiated the validation of selected XRN2-associating proteins of interest, but it is important to note that validation of a large number of novel protein partners and investigation of underlined biological significance of these interactions are warranted. Interestingly, this approach guided us to investigate the complexity of phenotypes that arise after XRN2 depletion. Such lines of investigations resulted in discovering the elevated PARP1 activity in XRN2 depleted cells and their sensitivity to PARP1 knockdown/inhibition (Figs. 3, 4, 5, and 7). XRN2 alterations including copy number variation, expression changes, and mutations are frequent in variety of cancers (Fig. 7). Some of these alterations could compromise XRN2 function and might render these cancers susceptible to PARP1 inhibition. Data presented here strongly suggest that XRN2 deficiency can be exploited by PARP1 inhibition.

## Methods

### Cell lines, cell culture, transfections, and siRNAs

Human HeLa, A549, HEK 293, and 293 T cells were obtained from the American Type Culture Collection (ATCC, Manassas, VA) and cultured in DMEM supplemented with 5% FBS and 1 mM l-glutamine in a humidified 5% CO_2_-95% air atmosphere at 37 °C. The shScr and shXRN2 immortalized human fibroblast cells were generated and validated as described previously^13,20^. Transfections were performed using Lipofectamine 2000 or RNAiMax (Life Technologies, Grand Island, NY) according to the manufacturer’s recommendation. Puromycin (1–2 µg/mL) was used 24 h after transfection to select for stable transfectants. Resistant clones derived from pooled populations were established in ~ 2 weeks. Limiting dilution assays were used to isolate individual clones, and knockdowns or over-expressions of TAP-XRN2 protein were validated using Western blotting.

Unless otherwise stated, all the siRNAs were obtained from Sigma-Aldrich (St. Louis, MO). For transient transfections, OptiMEM, RNAiMax, siScr, siXRN2 pool (siRNA#1, from Santa Cruz Biotechnology (Dallas, TX)) or 3′-UTR specific-siXRN2 (siRNA#2), and indicated siRNAs were used. Typical transfection experiments were done in 6-well plates (200,000 cells/well) using 25 nM siRNAs for 48–72 h. For experiments describing cell survival after addition of PARP1 inhibitors, exposure to PARP1 inhibitors was completed within 48–72 h of transfection.

### TAP constructs, TAP-XRN2 purification, and mass spectrometric analyses

The Xrn2 cDNA was obtained from OriGene (Rockville, MD). The pIRES-puro-TAP plasmid (CloneTech, Mountain View, CA) was used as a TAP-empty vector control. To clone pIRES-TAP-XRN2 construct, Xrn2 gene primers (harboring *FseI* and *AscI* restriction sites) and Xrn2 cDNA (as a template) were used. These vectors were then used to generate stable cell lines. For the TAP-XRN2 purification, several modifications were made to the originally described procedure and the method employed is essentially the same as TAP-K-H purification described in details elsewhere^24,44,45^. Eluted proteins were concentrated using YM-3 centrifugal filters (EMD Millipore, Billerica, MA), quantified using BCA assay, analyzed by SDS-PAGE, and used for mass spectrometric analyses.

Mass spectrometric analyses were performed as described previously^24^. Initial lists obtained from mass spectrometric analyses were further filtered requiring distinguished peptide identity (i.e., indistinguishable proteins were filtered out), peptide sequences ≥ 5, PSM ≥ 5, % coverage ≥ 5, either exclusively present in the TAP-XRN2 fraction or enriched in TAP-XRN2 pull-downs (TAP-XRN2/TAP ratio > 1.0). Identified proteins were presented by their UniProt accession number along with the above-mentioned information. The list reported here represents three biological replicates (Table S1). Proteins fitting all the above-mentioned criteria were included in subsequent evaluations.

### Bioinformatics analyses

Procedure for bioinformatics analyses is similar as described previously^24^. For functional annotation and biological mechanisms analyses, DAVID v6.7 (Database for Annotation, Visualization and Integrated Discovery, (https://david.abcc.ncifcrf.gov/)) bioinformatics tool was used that allowed us to further scrutinize the proteins identified in mass spectrometric screening^46,47^. For DAVID analysis, the default setting was used, and the bar graph presented is based on the FDR cutoff 0.05.

For identification of canonical pathways associated with XRN2, Ingenuity Pathway Analysis (IPA) was employed with using proteins identified in mass spectrometric screening as input. We performed core analysis using the Ingenuity knowledge base as the reference set. Fisher’s exact test identified top canonical pathways that were significantly enriched among these proteins. To generate protein–protein interaction network, STRING 9.1 (Search Tool for the Retrieval of Interacting Genes/Proteins, (https://string-db.org)) analyses were carried out using default setting^48^. The protein–protein interaction prediction methods used were based on neighbourhood, gene fusion, co-occurrence, co-expression, experimental data, databases, and text mining. The protein interaction layout was created by Cytoscape 3.4.0 software (https://cytoscape.org) using the compatible input file from STRING^49^.

### Western blot analyses

Procedure for Western blotting is similar as described previously^24^. Briefly, proteins were separated by SDS-PAGE and transferred to PVDF membranes. Blots were treated with 1× blocking buffer (Sigma, St. Louis, MO) and incubated with primary and appropriated secondary antibodies conjugated with HRP. Proteins were detected by enhanced chemiluminescence HRP substrates, Super Signal Pico or Dura (Thermo Fisher Scientific, Waltham, MA). For western blot quantification, intensities of protein bands were analyzed using NIH ImageJ software and specific protein band intensities were normalized to α-tubulin loading control. The reported relative levels are the results of n ≥ 3. Raw data for all the Western blot images are provided in the supplementary information. An antibody against α-tubulin (DM1A) was purchased from Sigma-Aldrich (St. Louis, MO). The antibodies against PARP1 (F-2) and XRN2 (H-3) were obtained from Santa Cruz Biotechnology (Dallas, TX). An antibody against XRN2 (Ab 72181) was also purchased from Abcam (Cambridge, MA). PAR antibodies (4335-MC) were obtained from Trevigen, (Gaithersburg, MD, USA).

### Co-immunoprecipitation (co-IP)

Procedure for co-immunoprecipitation is similar as described previously^24^. HEK 293 T TAP-XRN2 cells (~ 3 × 10^6^) were seeded onto 150 mm dishes and allowed to grow until ~ 80% confluency. Cells were then harvested and whole-cell lysates were prepared by re-suspending the pellet in 1 mL 1× TNE buffer (100 mM Tris–HCl (pH 7.5), 150 mM NaCl, 0.1 mM EDTA, and 0.1% NP-40 (v/v) supplemented with 0.5 mM PMSF, 1× protease inhibitor cocktail and 10 mM β-glycerophosphate). Cell lysates were sonicated and centrifuged (10,000*g* for 15 min, 4 °C), supernatants were collected, and total protein was quantified using BCA assays (Thermo-Scientific, Waltham, MA). A portion of each sample was saved as input. For co-IP analyses, ~ 1 mg protein was incubated with A/G plus agarose beads (Santa Cruz Biotechnology, Dallas, TX) for control or 10 µg anti-XRN2 antibodies. Mixtures were then incubated overnight at 4 °C, collected by centrifugation (1,000*g*, 5 min, 4 °C), washed (3×) with 1× PBS buffer, re-suspended in Laemmli buffer (Bio-Rad, Hercules, CA), and heated for 5 min at 95 °C. Proteins in the supernatants were separated by SDS-PAGE and analyzed by Western immunoblotting using various specific antibodies as indicated. For co-IP using recombinant XRN2 and PARP1, identical procedure was employed as mentioned above except purified proteins (5 µg each) were used instead of cell lysate. Purified XRN2 was obtained from OriGene Technologies Inc (Rockville, MD) and Flag-PARP1 was a generous gift from Chen-Ming Chiang laboratory at UT Southwestern (Dallas, TX).

### Gel filtration chromatography

Procedures for HeLa whole-cell lysate preparation, nuclease treatment (to eliminate nucleic acids), and gel-filtration chromatography were essentially the same as described previously^13^.

### Cell survival analyses

A modified cell survival assay that measures DNA content over a 7-day period was employed^50^. A stock concentration of BMN (Selleckchem, Houston, TX) was prepared in DMSO and stored at − 80 °C. For cell survival assays using BMN, shScr fibroblast cells (20,000 cells/well) transiently transfected with siScr or siXRN2 were seeded into 48-well plates in 0.5 mL media. The next day, cells were exposed to BMN for 24 h, the media was then removed, cells were washed with 1× PBS, and fresh media was added. Cells were then allowed to grow until control samples reached confluency (i.e., ~ 7 days). For cell survival assays related to focused siRNA screening, cells were allowed to grow for 72 h after siRNA addition. Cells were then lysed in water, freeze-thawed, and DNA content (a measure of cell growth) was determined by Hoescht 33258 fluorescence (Sigma, St. Louis, MO) using a Victor X3 plate reader (PerkinElmer, Waltham, MA). Data (mean ± SEM) were expressed as treated/control (T/C) values from experiments performed at least three times in triplicate each and *p*-values were obtained using two-tailed student’s t-tests.

### Clonogenic survival analyses

The A549 cells treated with siScr or siXRN2 #1 were seeded on 60-mm dishes. The next day, cells were treated for 24 h with indicated concentrations of PARP1 inhibitor BMN (µM) or vehicle (0.01% DMSO), the media was then removed, cells were washed with 1× PBS, and fresh media was added. Cells were then allowed to grow for 7 days. Then, the media was removed; cells were washed with 1× PBS, and stained with crystal violet solution. Colonies (with > 50 normal-appearing cells) were counted and data (mean ± SEM) were expressed as treated/control (T/C) values from experiments performed at least three times in triplicate and *p*-values were obtained using two-way ANOVA with Sidak's multiple comparisons test.

### Immunofluorescence confocal microscopy

Cells were seeded on 6-well plates (~ 200,000 cells/well) containing glass slides and allowed to adhere overnight. The next day, cells were treated with siScr or siXRN2 #1 (25 nM) for 48 or 72 h with co-treatment of DMSO or 10 nM PARP1i (BMN 673, dissolved in DMSO) for the last 24 or 48 h, respectively. For positive control, cells were treated with 1 mM H_2_O_2_ for 15 min. Then, cells were fixed by gentle washing in 1× PBS, followed by fixation with ice-cold methanol:acetic acid (3:1, v/v) overnight at − 20 °C. Fixed cells were gently washed in 1× PBS at room temperature (3×, 5 min each). The cells were then incubated in blocking solution (5% normal goat serum in 1× PBS) for 1 h at room temperature. Then, the cells were incubated with PAR or γH2AX primary antibody (1:1,000 and 1:2,000 dilution, respectively, in 1× PBS containing 5% normal goat serum) for 3 h at room temperature. Then, the cells were washed (3×, 5 min each in 1× PBS + 0.1% Tween 20) and incubated with Alexa Fluor 594 or 488 fluorescent secondary antibody (1:1,500 and 1:2,000 dilution, respectively, in 1× PBS containing 5% normal goat serum) for 1 h at room temperature. The cells were then washed (3×, 5 min each in 1× PBS + 0.1% Tween 20). Finally, the wash buffer was removed, and the cover glass was mounted with prolong gold antifade mounting medium containing DAPI (nuclear stain). Images were obtained using Olympus FV10i confocal laser scanning microscope with 60 × oil immersion objective. Images were processed using ImageJ. The nuclei of 75 individual cells were analyzed and fluorescence intensity of PAR or γH2AX for different treatment groups (i.e., siRNA ± DMSO/BMN 673/H_2_O_2_) were obtained then normalized to siScr for PAR and γH2AX, respectively. Next, an ordinary one-way ANOVA (95% confidence interval (alpha = 0.05) was performed comparing siXRN2 #1, siXRN2 #1 + PARPi, and the H_2_O_2_ positive control means to the siScr control or siXRN2 #1 to siXRN2 #1 + PARPi.

### Statistical analyses

Where appropriate, data are represented as mean ± SEM from a minimum of three experiments. Unless stated otherwise, *p*-values were obtained using two-tailed student’s t-tests.

## Supplementary information

Supplementary Information

Supplementary Table S1.

Supplementary Table S2.

Supplementary Table S3.

## Abbreviations

BER: Base excision repair
Co-IP: Co-immunoprecipitation
DAVID: Database for Annotation, Visualization and Integrated Discovery
DSBs: DNA double strand breaks
FDR: False discovery rate
HR: Homologous recombination
IPA: Ingenuity Pathway Analysis
MS: Mass spectrometry
NHEJ: Non-homologous end joining
PAR: Poly(ADP-ribose)
PMSF: Phenylmethylsulfonyl fluoride
STRING: Search Tool for the Retrieval of INteracting Genes/proteins
TAP: Tandem affinity purification
