# Supplementary material for: XRN2 interactome reveals its synthetic lethal relationship with PARP1 inhibition
Source: Sci Rep. 2020 Aug 28;10:14253. doi: 10.1038/s41598-020-71203-7 (PMC7455564; doi:10.1038/s41598-020-71203-7)

Supplementary Data

XRN2 interactome reveals its synthetic lethal relationship with PARP1 inhibition

Praveen L. Patidar[1]*, Talysa Viera[1], Julio C. Morales[2], Naveen M. Singh[3], Edward A. Motea[3], Megha Khandelwal[1], and Farjana J. Fattah[4]

[1]Department of Chemistry, New Mexico Institute of Mining and Technology, Socorro, NM, 87801, USA.
[2]Department of Neurosurgery, University of Oklahoma Health Science Center, Oklahoma City, OK 73104, USA.
[3]Department of Biochemistry and Molecular Biology, Simon Cancer Center, Indiana University School of Medicine, Indianapolis, Indiana, 46202, USA.
[4]Simmons Comprehensive Cancer Center, University of Texas Southwestern Medical Center, Dallas, TX 75390, USA

* To whom correspondence should be addressed: Praveen Patidar, Department of Chemistry, New Mexico Institute of Mining and Technology, 801 Leroy Pl., Socorro, NM, 87801, USA; *Praveen.Patidar@nmt.edu;* Phone: +1 575 835 5007, Fax: +1 575 835 5364

Materials included-

1. This file with supplementary table legends and figures
2. Supplementary tables S1, S2, and S3 in excel file format

FIGURES LEGENDS

Table S1. **List of proteins identified by mass spectrometric analysis of TAP-XRN2 pull-down.** Proteins with distinguished peptide identity, peptide sequences ≥5, PSM ≥5, % coverage ≥5, either exclusively present in the TAP-XRN2 fraction or enriched in TAP-XRN2 pull-downs (TAP-XRN2/TAP ratio >1.0) are shown. This list represents known and novel XRN2-associating proteins. Proteins are represented by their UniProt accession ID and protein description. Also included are PSMs (number of spectra assigned to peptides that contributed to inference of a protein), peptide sequences (number of different unique peptide sequences, or modified variants of sequences identified for the protein), % sequence coverage (percentage of protein sequence covered by peptides identified for a specific protein), spectral index (MIC Sin), and enrichment ratio (TAP-XRN2/TAP ratio). TAP-XRN2 purification and mass spectrometric analyses were representative of three biological replicates.

Table S2. DAVID analyses for functional annotation of proteins associated with XRN2. Functional categories determined by DAVID v6.7 for proteins identified in **Table S1**. The top five functional categories with respective population (Pop) hit values and *p*-values are shown.

Table S3. Ingenuity Pathway Analysis (IPA) to identify XRN2-linked canonical pathways. Proteins indicated in **Table S1** were used as inputs in IPA to identify XRN2-linked canonical pathways. The top five canonical pathways linked to XRN2 with respective log [*p*-values] and molecules are shown.

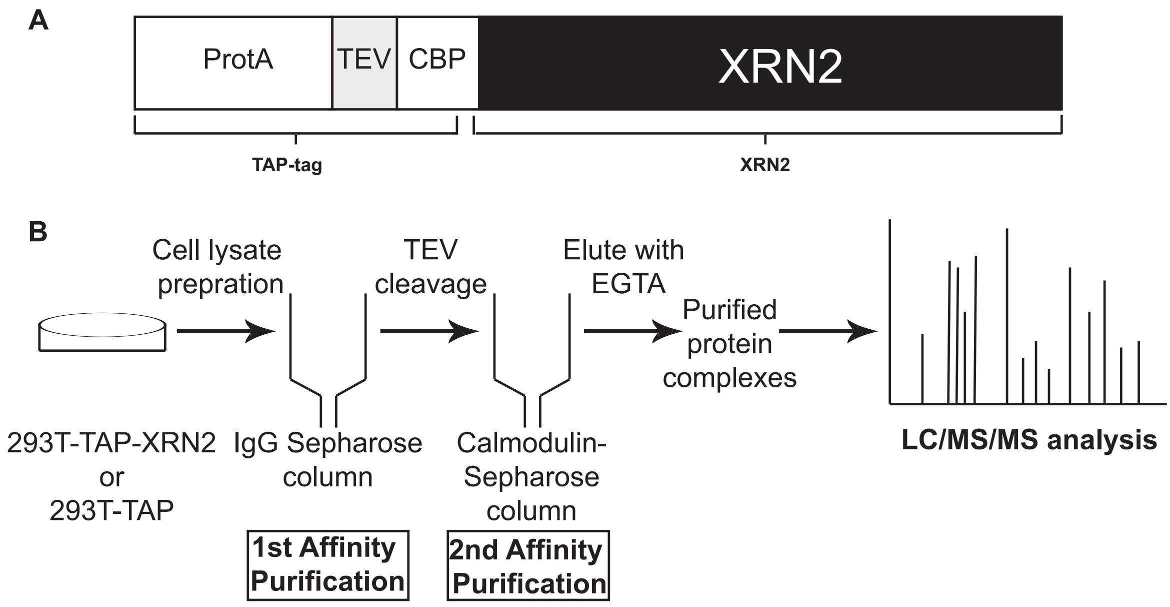

Figure S1. Outline of TAP-XRN2 purification and identification of associating proteins. **(A)** Schematic representation of the N-terminal TAP-tag fused to XRN2 protein. The TAP-tag is composed of two immunoglobin G (IgG) binding domains of Protein A (ProtA) from *Staphylococcus aureus* and a Calmodulin binding peptide (CBP) separated via a linker that is cleavable by TEV protease. **(B)** General outline of the TAP purification strategy to identify XRN2-associting proteins. Stable clones of 293T cells containing TAP or TAP-XRN2 constructs were generated and large-scale cultures of these cells were prepared. Cells were lysed in lysis buffer by repeated freeze-thaw cycles followed by sonication to release the soluble proteins. Cell debris was removed by centrifugation and the soluble fraction was subjected to two sequential steps of affinity purification. Complex mixtures of purified TAP or TAP-XRN2 along with associating proteins were examined by mass spectrometric analyses.

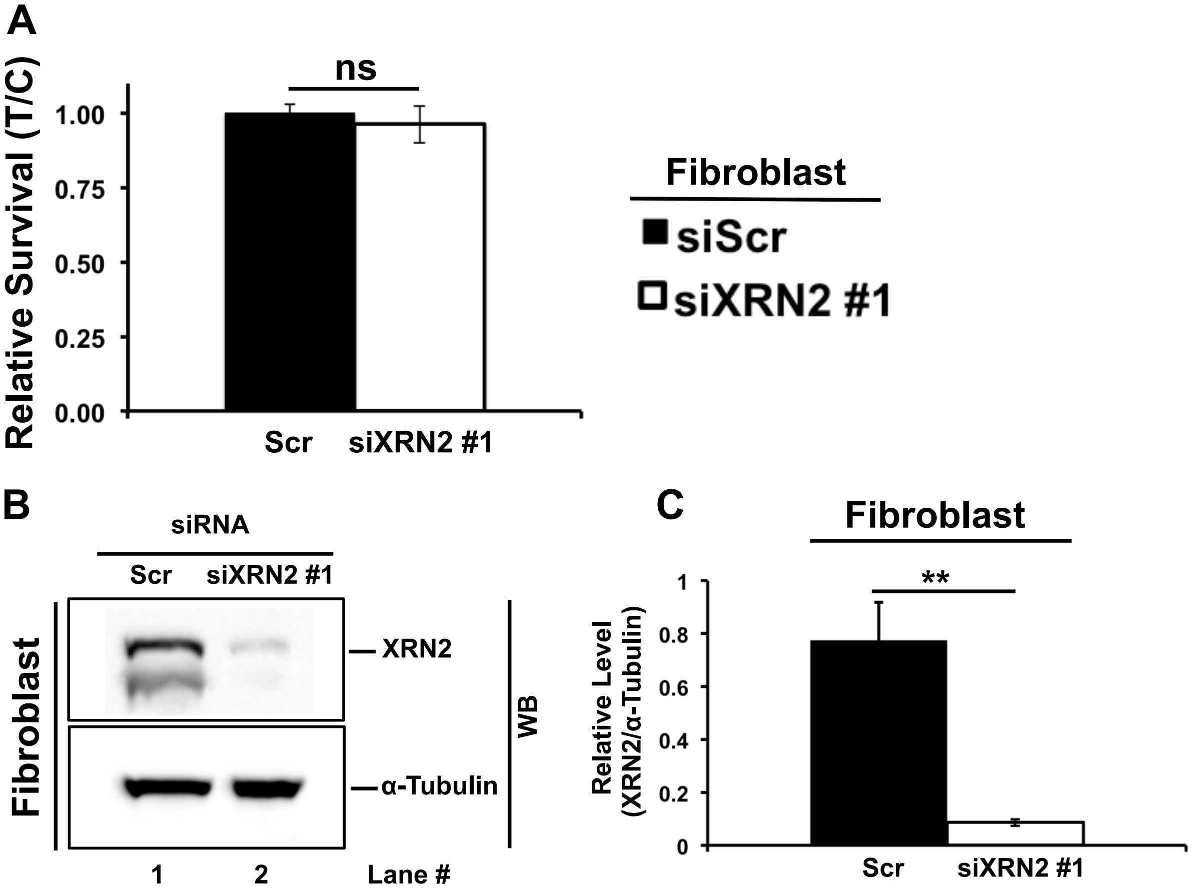

Figure S2. Transient knockdown of XRN2 does not affect cell survival. (A). Relative cell survival analyses of fibroblast cells after transient (72 h total) siScr (control) or siXRN2 #1 knockdown. Cell survival analyses were carried same as described for figure 3 and 5. Graph represent mean ± SEM for treated (i.e., siRNA/control (T/C) samples from experiments performed 4 times (each in triplicate). *p*-values were obtained via two-tailed student's t-tests. ns, not significant comparing siScr *vs.* siXRN2. Note that transient knockdown of XRN2 does not lead to significant change in cell survival. **(B-C)** Representative Western blot image (B) and quantification (C) showing successful knockdown of XRN2 (72 h after transfection) in cells that were used in panel A. As a loading control, α-tubulin was used.

Figure 2B
supp.

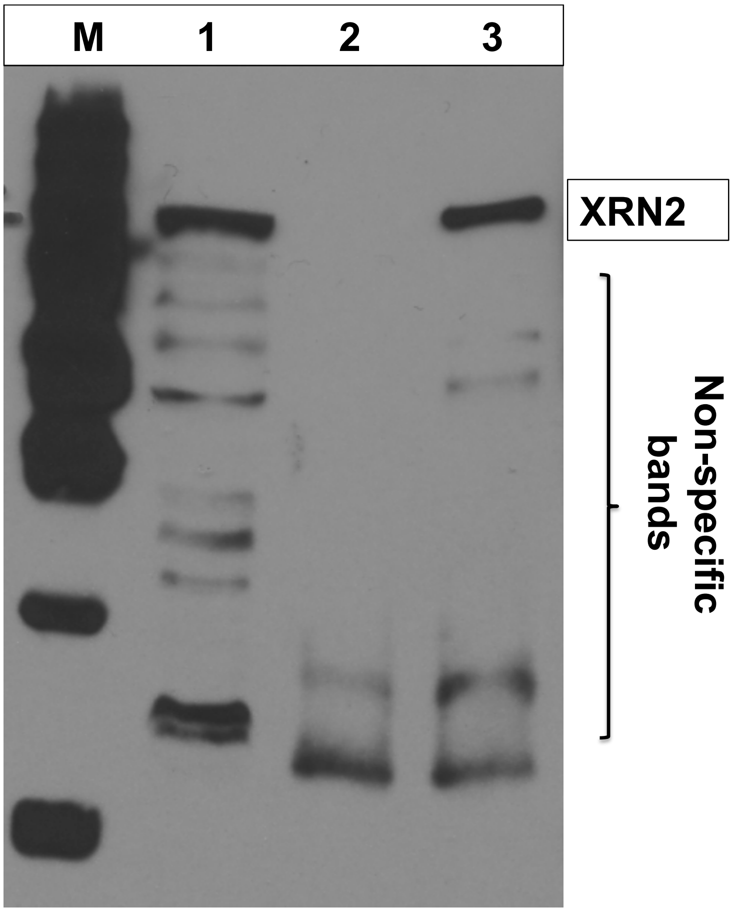

Figure 2B supp.

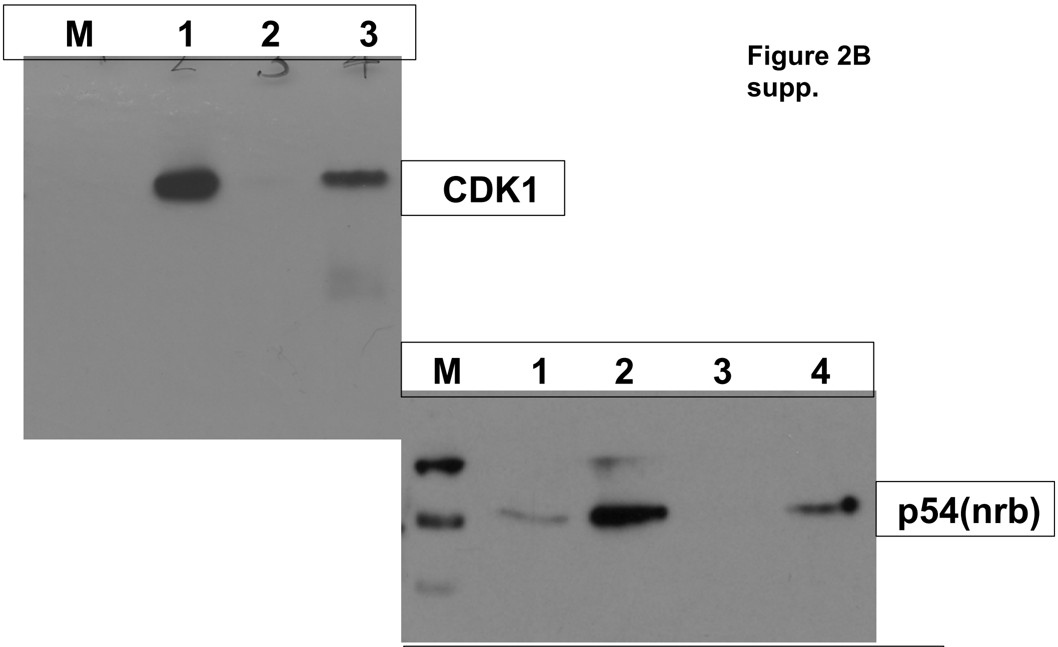

Lane 1 is lower quantity of input

Figure 2E & F supp.

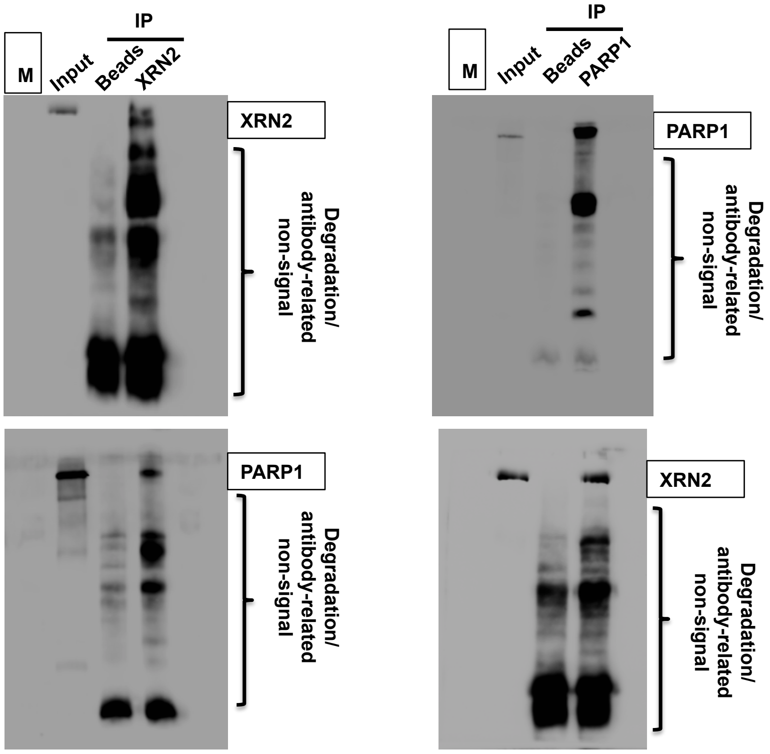

Molecular weights were matched by superimposing the marker lane on the imager during image capture.

Figure 2G supp.

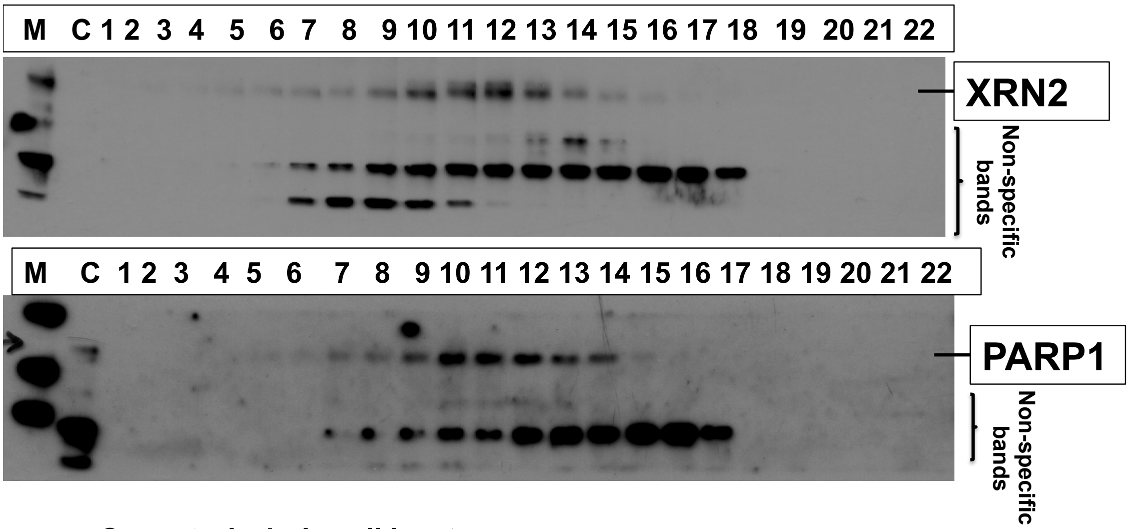

C: control whole cell lysate

Figure 4C supp.

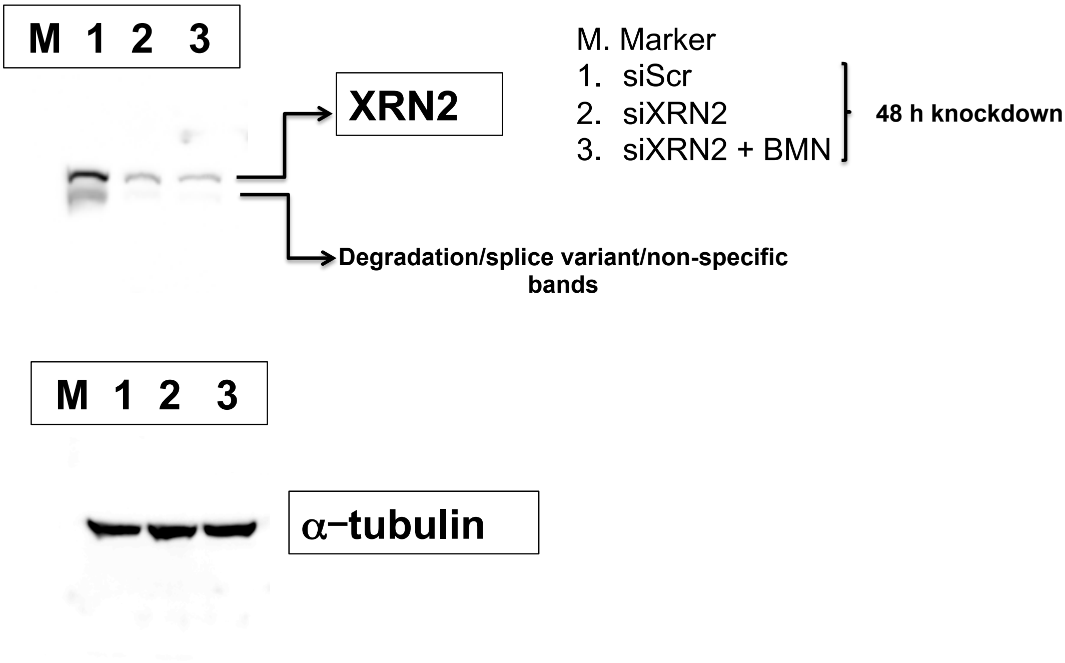

Figure 4G supp.

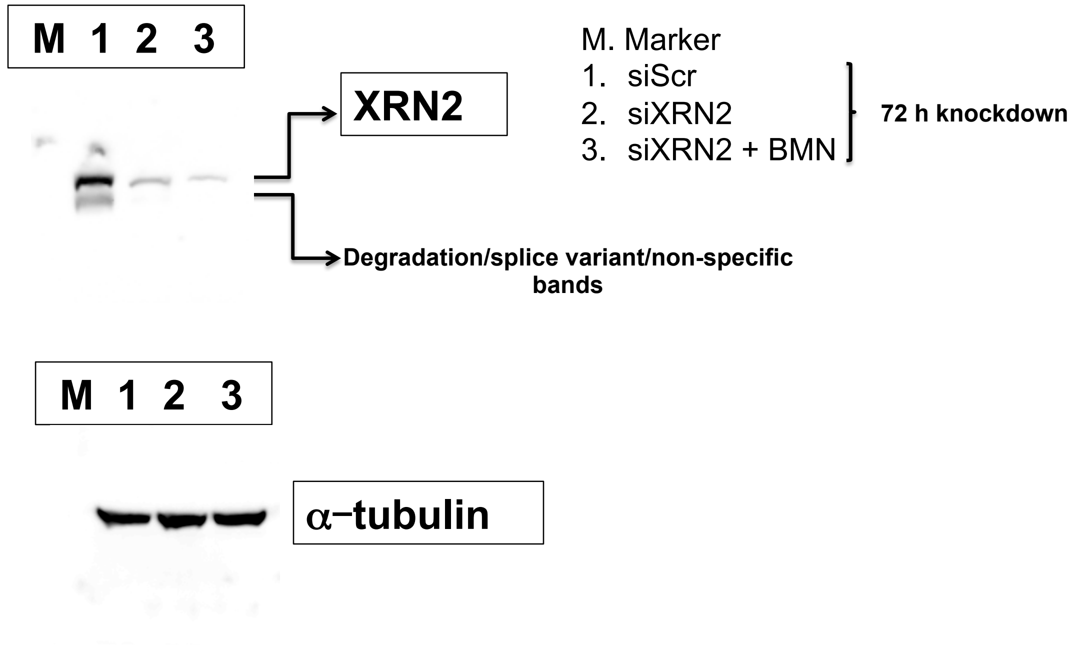

Figure 5 supp.

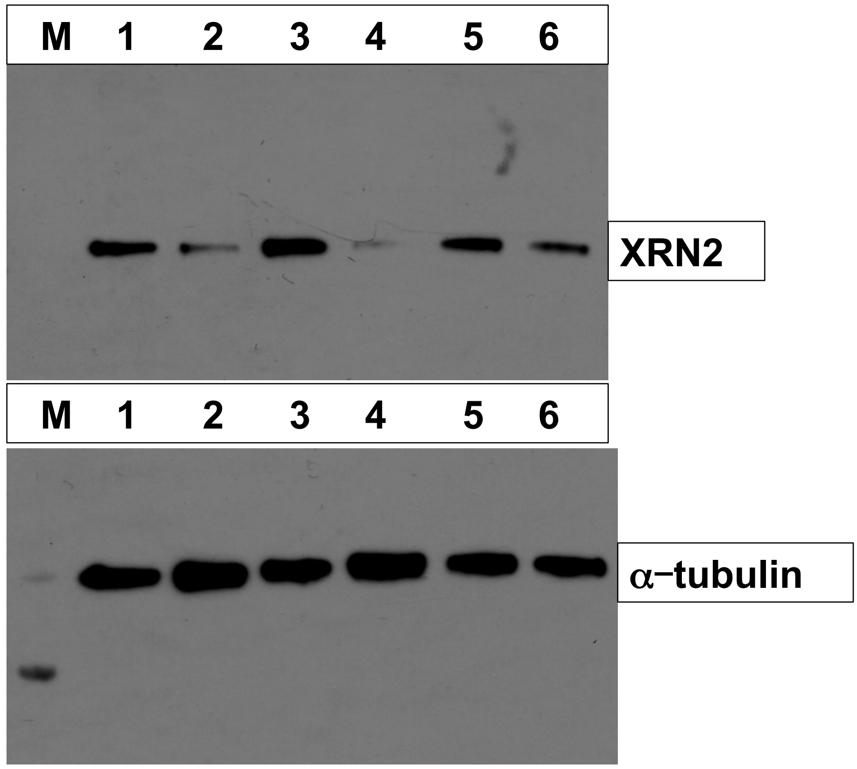

Lanes 3-4 are unrelated samples, only lanes 1-2 (fig. 5A) and lanes 5-6 (fig. 5B) were used.

Figure 6C supp.

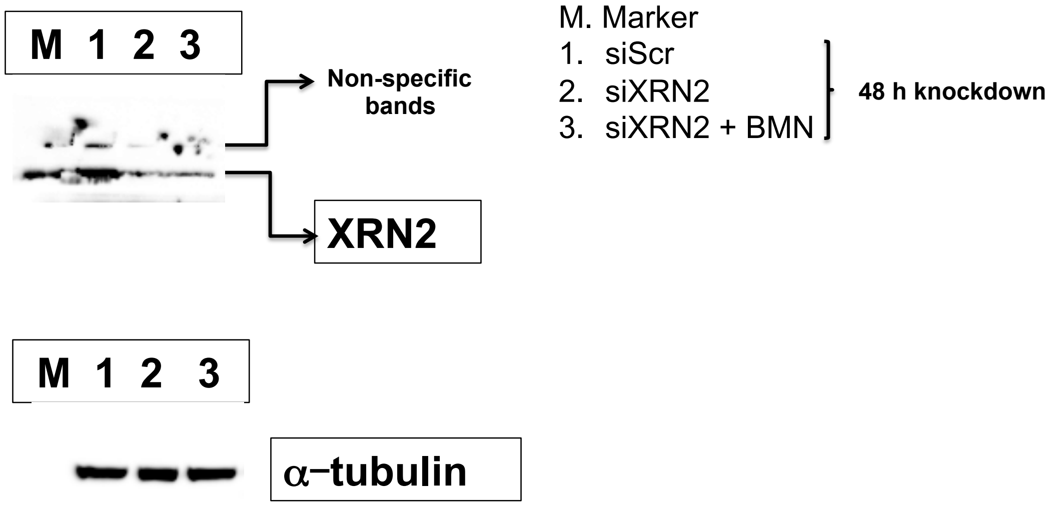

Figure 6C supp.

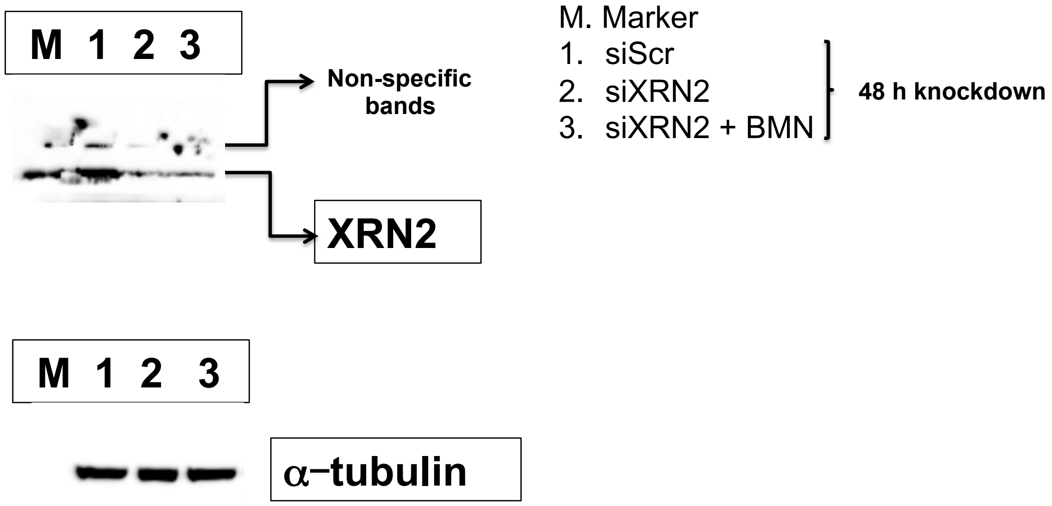

Figure 7E supp.

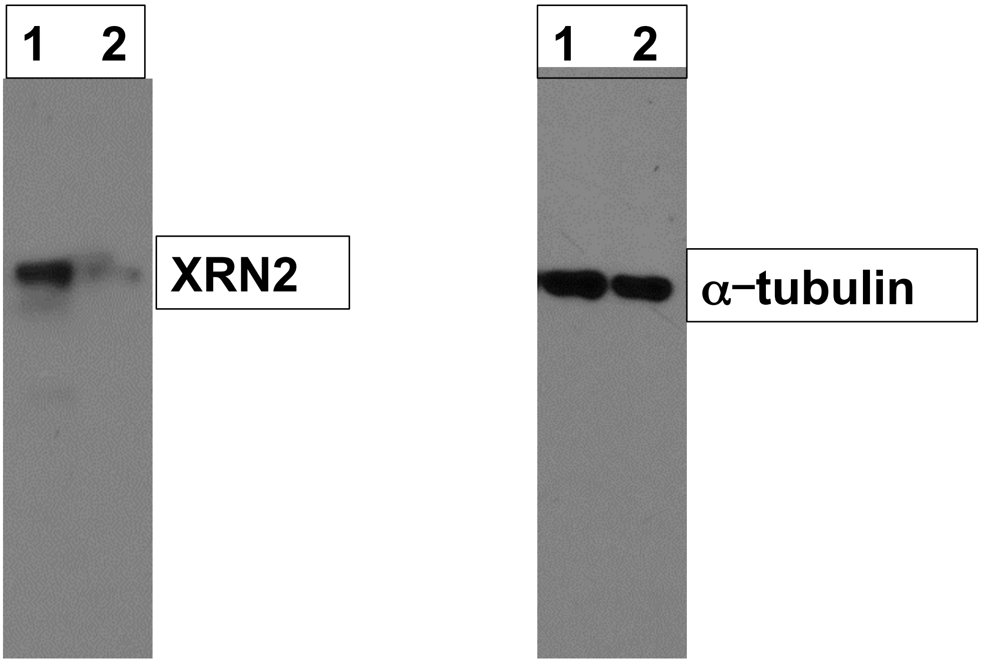

Figure S2B supp.

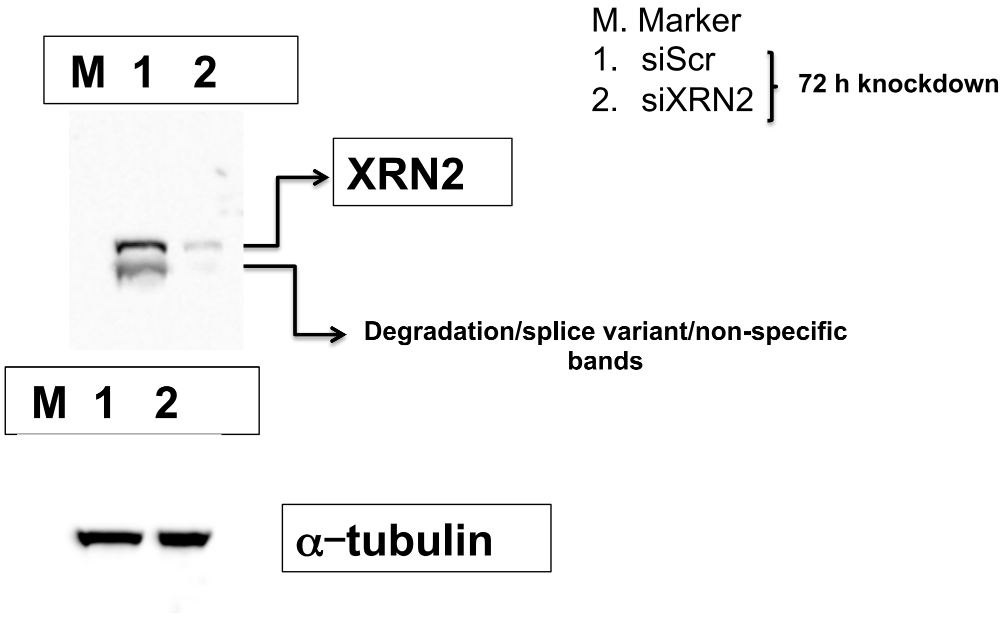

Supplement: Supplementary file 1 — Supplementary Information [file 41598_2020_71203_MOESM1_ESM.pdf]
